# iCREATE: imaging features of primary and metastatic alveolar soft part sarcoma from the EORTC CREATE study

**DOI:** 10.1186/s40644-020-00352-9

**Published:** 2020-10-30

**Authors:** Naami Charlotte Mcaddy, Hind Saffar, Saskia Litière, Pieter Jespers, Patrick Schöffski, Christina Messiou

**Affiliations:** 1 Department of Radiology, The Royal Marsden Hospital, London, UK; 2 Soft Tissue and Bone Sarcoma Group, European Organisation for Research and Treatment of Cancer Headquarters, Brussels, Belgium; 3 Department of General Medical Oncology and Department of Oncology, KU Leuven, Leuven Cancer Institute, University Hospitals Leuven, Leuven, Belgium; 4 The Institute of Cancer Research, London, UK

**Keywords:** Alveolar soft part sarcoma, Soft tissue sarcoma, Metastatic disease, Computer tomography

## Abstract

**Background:**

Alveolar Soft Part Sarcoma (ASPS) is a rare, slow-growing, but highly vascular soft tissue sarcoma, characterised by a high rate of metastases at presentation. Although imaging features of the primary are well described, less detail is available on the imaging pattern of metastatic ASPS. The EORTC 90101 (CREATE) study assessed the efficacy of Crizotinib in patients with metastatic ASPS and presents a unique opportunity to describe the imaging phenotype of primary and metastatic ASPS, based on prospectively collected imaging.

**Methods:**

A retrospective review of the staging CT scans of 32 patients with ASPS from the CREATE study was undertaken and the imaging features of primary and metastatic disease were assessed.

**Results:**

Imaging of the primary tumour was available in 7/32 cases (28%). All primary tumours demonstrated marked vascularity with prominent feeding vessels (7/7, 100%). The most frequent sites of metastases included lung (30/32, 94%), nodal (7/32, 22%), bone (5/32, 16%) and muscle/subcutaneous (5/32, 16%). Features of hypervascularity were identified at all sites, more appreciable in the lungs, with feeding vessels frequently demonstrated in pulmonary metastases (21/32, 66%).

**Conclusion:**

Analysis of imaging from the CREATE cohort of patients with metastatic ASPS demonstrates that metastases from ASPS are predominantly hypervascular and demonstrate feeding vessels comparable to primary ASPS, suggesting potential sensitivity of this rare sarcoma for antivascular/antiangiogenic treatment approaches.

## Introduction

Alveolar Soft Part Sarcoma (ASPS) is a rare soft tissue tumour accounting for less than 1% [1] of soft tissue sarcomas*.* It most commonly occurs in younger patients between 15 and 35 years with a slight female preponderance [2]. It frequently arises in the lower extremities [3] but may also arise from the trunk and retroperitoneum and, in younger children, the orbit and tongue [4]. It is a relatively slow growing tumour, associated with minimal clinical symptoms [5] characterised by a high rate of metastases at presentation [6].

Imaging is essential for assessing the primary tumour and extent of metastatic spread. Whilst the radiological features of primary ASPS have been described with computer tomography (CT) and magnetic resonance (MR) in serial case reports, comparatively less information is available on the imaging characteristics of the metastases. The European Organisation for Research and Treatment of Cancer (EORTC) recently conducted a prospective, multi-centre Phase II trial (EORTC 90101; NCT01524926- Cross-tumoral Phase 2 with Crizotinib, CREATE) evaluating crizotinib in an independent cohort of patients with locally advanced and metastatic ASPS [7]. The baseline imaging data (iCREATE) from these centrally confirmed ASPS cases presents a unique opportunity to describe the imaging features of metastatic disease from this very rare sarcoma subtype.

## Material and methods

### Patient selection

The original CREATE cohort of patients with centrally confirmed histological diagnosis of ASPS consisted of 53 patients. The full protocol with study inclusion and exclusion criteria related are available as part of the published CREATE trial manuscript [7]. Imaging performed immediately prior to study entry (baseline images) were unavailable from the referring institutions for 15 (28%) of these patients. The images for the remaining 38 (72%) patients were submitted from the EORTC to the Royal Marsden for evaluation. A minimum imaging dataset of a contrast-enhanced CT scan of the thorax, abdomen and pelvis was required for inclusion. Thirty-two (60%) patients fulfilled this criterion and were included in the iCREATE imaging database.

### Image evaluation

The anonymised CT images of patients from the CREATE database were uploaded onto our institution’s Picture Archiving and Communication System (PACS) and held on a protected server for the duration of the review. Images were reviewed independently by two radiologists and the imaging case report forms (CRFs) identifying the relevant radiological features were completed. Evaluated parameters of primary and metastatic disease included site, size, presence of increased vascularity, feeding vessels, calcification, necrosis, haemorrhage and irregular margins. Hypervascularity was defined relative to skeletal muscle. Assessment of metastatic bone disease assessment was limited to documentation of phenotype (lytic, sclerotic, mixed) and presence/absence of a soft tissue component.

### Statistical analysis

Categorical data were summarized using frequency and percentage. For continuous variables the range and median were reported. (IBM SPSS Statistics for Windows, IBM, Armonk, USA).

## Results

### Patient demographics

The iCREATE cohort consisted of 14 females and 18 males, with a median age of 30 years (range: 16–69 years; interquartile range 24–37 years).

### Imaging of primary ASPS

**Fig. 1:**
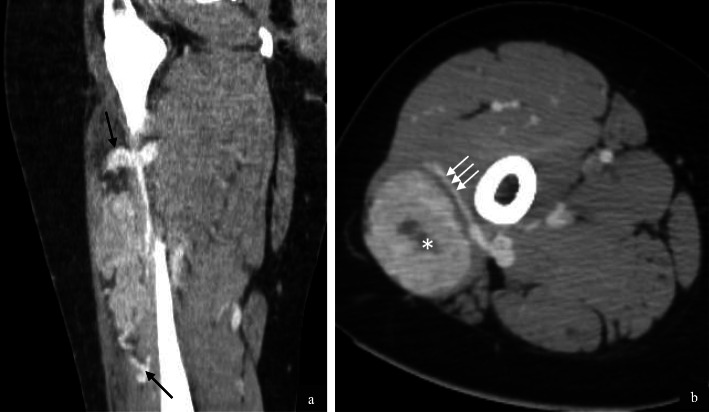
Intramuscular lower limb primary ASPS in a 16 year old female patient. Post contrast coronal (**a**) and axial (**b**) CT images demonstrate hypervascularity of the primary tumour. Feeding vessels at the upper and lower poles (**a**, black arrows) as well as circumferential vessels (**b**, white arrows) are present. Small volume necrosis is seen centrally (**b**, white asterisk)

**Fig. 2:**
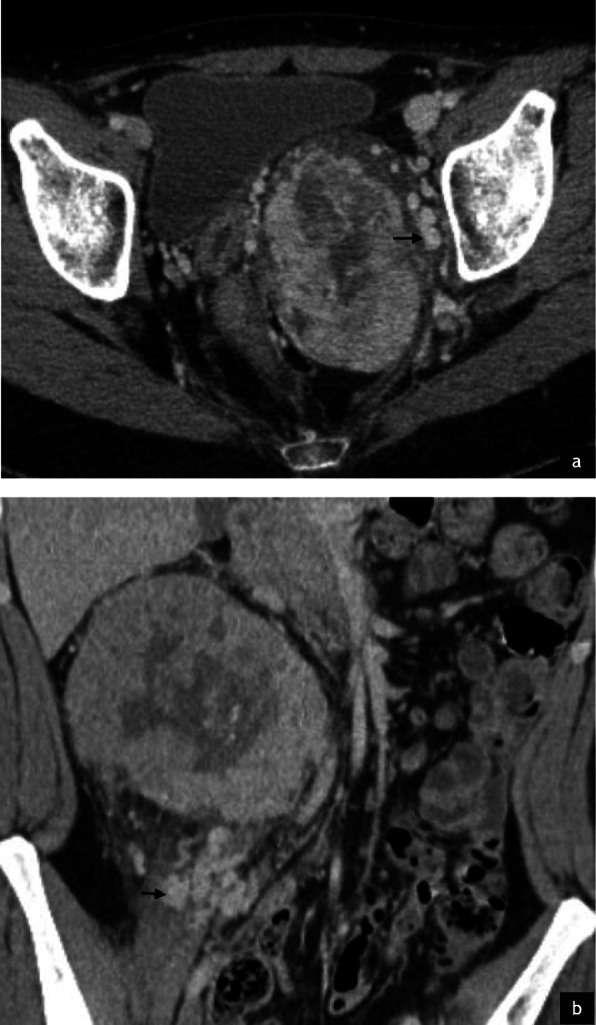
Primary ASPS within the pelvis in a pelvic 22 year old female (**a**) and intra-abdominal primary ASPS in a 24 year old male (**b**) patient. Post contrast axial (**a**) and coronal (**b**) CT demonstrates large tortuous feeding vessels at the periphery of both tumours (black arrows) with marked tumour hypervascularity. Although there is significant mass effect, both masses demonstrate well defined borders with no evidence of local invasion

Imaging of the primary tumour on CT was available for 7/32 (22%) patients. Primary sites included the trunk (2/7), retroperitoneum/pelvis (1/7) and extremities (4/7). Mean tumour size was 6.4 cm (range 1.7–14.0 cm). All primary tumours demonstrated increased vascularity with tortuous feeding vessels (Figs. 1, 2). Necrosis was present in 5/7 cases (Fig. 2). Well-defined tumour margins were demonstrated in 4/7 primary tumours. Calcification in the primary mass was demonstrated in 1/7 cases. Haemorrhage was not of a feature of any of the primary masses imaged.

### Imaging of metastatic ASPS lesions

As per inclusion criteria of the CREATE trial, all patients within our cohort had at least one site of metastasis. A single site was confirmed in 16/32 patients, 11/32 patients had two sites, 2/32 patients had three sites, 2/32 patients had 4 sites and 1/32 patient had 5 sites. Table 1 outlines the sites and imaging features of metastatic disease.

**Table 1:** Summary of imaging features of metastatic ASPS lesions

***Total Number of patients, n = 32***	N (%)	Number of lesions			Size range, (mean) cm	Feeding vessel n (%)	Hyper-vascular n (%)	Well-defined n (%)	Haemorrhage n (%)	Necrosis n (%)	Calcification n (%)
		1 to 5	6 to 9	> 10							
***Site***											
Pulmonary	30 (94)	2	7	23	0.2–5.8 (1.8)	21 (70)	25 (83)	28 (93)	0 (0)	11 (37)	0 (0)
Brain/CNS	2 (6)				1.3–2.1 (1.7)	1 (50)	2 (100)	2 (100)	0 (0)	1 (50)	0 (0)
Visceral											
Liver	3 (9)	3			1.1–4.1 (2.2)	1 (33)	1 (33)	3 (100)	0 (0)	2 (67)	0 (0)
Spleen	3 (9)	3			1.5–5 (3.0)	0 (0)	0 (0)	3 (100)	0 (0)	3 (100)	0 (0)
Renal	2 (6)	2			2.1–3.5 (2.6)	0 (0)	1 (50)	2 (100)	0 (0)	1 (50)	0 (0)
Adrenal	2 (6)	2			2.4–3 (2.7)	0 (0)	1 (50)	2 (100)	0 (0)	2 (100)	0 (0)
Nodal											
Mediastinal	6 (19)	2	3		1.6–9.1 (3.1)	4 (60)	4 (67)	5 (83)	0 (0)	6 (100)	0 (0)
Pelvic	1 (3)	1			2.1 (2.1)	0 (0)	0	0 (0)	0 (0)	0 (0)	0 (0)
Subcutaneous	1 (3)	1			1.4 (1.4)	1 (100)	1 (100)	1 (100)	0 (0)	0 (0)	0 (0)
Intramuscular	4 (13)	4			1.4–4.3 (6.7)	4 (100)	4 (100)	4 (100)	0 (0)	0 (0)	0 (0)
Bone											
Metastases	5 (16)										
*Soft tissue*	3 (9)										
*Lytic*	2 (6)										
*Sclerotic*	1 (3)										
*Mixed*	2 (6)										

**Fig. 3:**
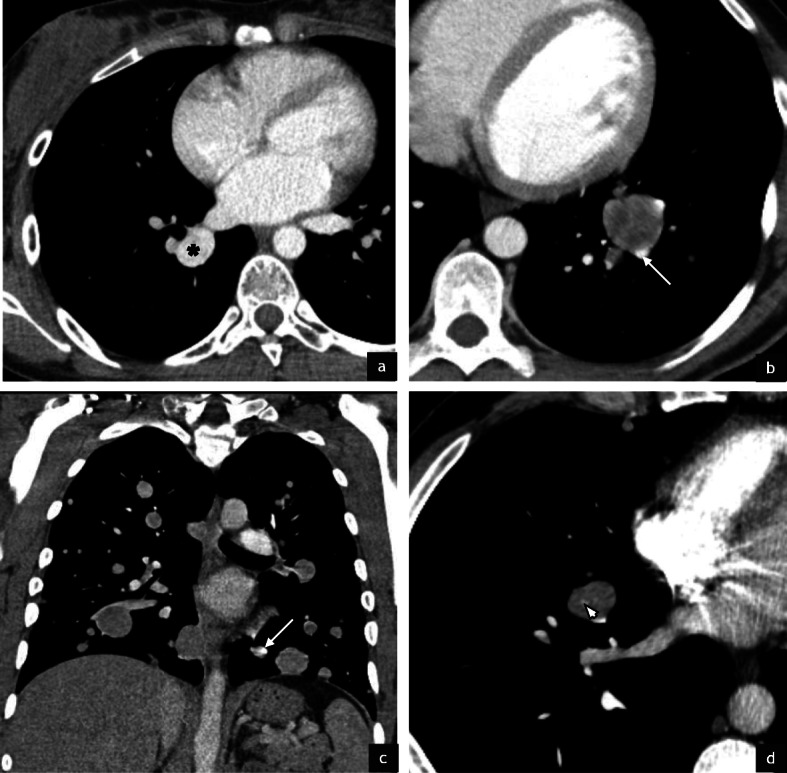
Pulmonary metastatic disease in ASPS. Post contrast axial CT in a 22 year old female (**a**) demonstrates a hyperdense right lower lobe pulmonary metastasis (**a**, black asterisk) which is almost isodense to the pulmonary vasculature. Post contrast CT in a 51 year old male (**b**) and a 29 year old male (**c**) shows prominent feeding vessels to multiple pulmonary metastases (**b**,**c** white arrows). Despite the small size of the metastases, intratumoural vessels can also be observed as in this post contrast CT of a 24 year old male patient (**d**, white arrowhead)

The most common metastatic site was the lung, with 30/32 patients (94%) having pulmonary metastases. These metastases demonstrated imaging features akin to those recognised in primary ASPS, namely, hypervascularity and prominent feeding vessels (Fig. 3). Haemorrhage and calcification were not observed in sites of metastatic disease.

**Fig. 4:**
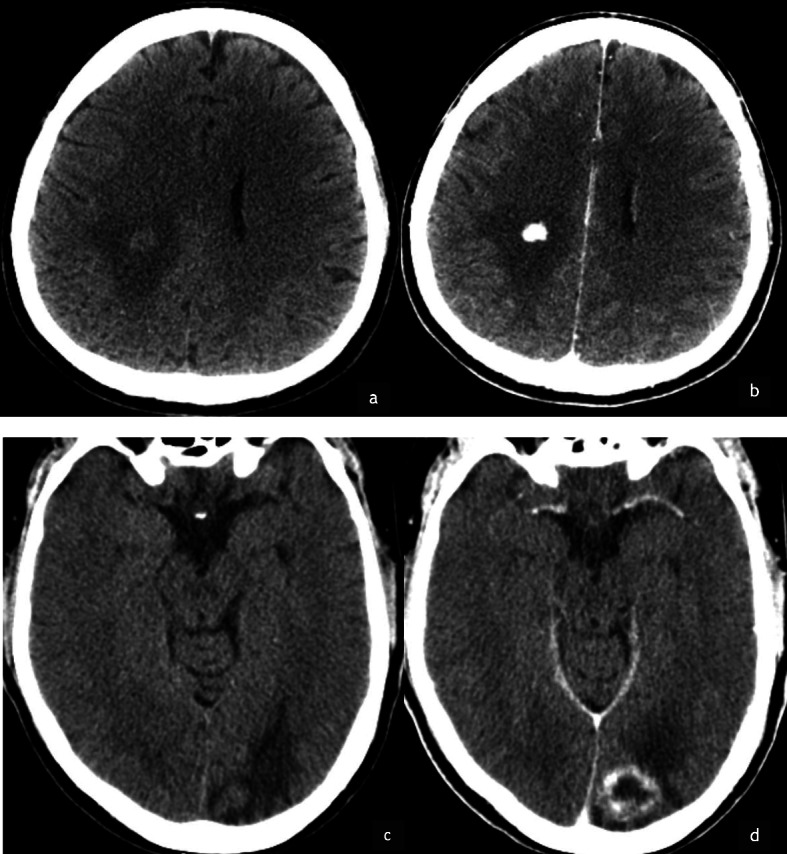
Pre and post contrast CT of the brain in a 33 year old female (**a** & **b**). White matter low attenuation is appreciated in the right parietal lobe in keeping with vasogenic oedema . Following contrast there is markedly avid enhancement (**b**) confirming intraparenchymal metastasis. Pre and post contrast axial CT of a 31 year old male (**c** & **d**) patient also demonstrates a similar morphology with avid peripheral enhancement of a left occipital lobe metastasis (**d**)

Intracranial imaging was not performed or not available for all patients. Within our cohort, brain metastases where identified in 2/32 patients (6%). Pre-and post contrast CT in these patients confirmed marked hypervascularity with little necrosis (Fig. 4).

**Fig. 5:**
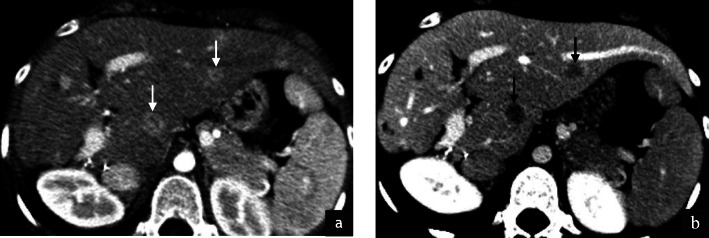
Hepatic metastases in a patient with alveolar soft part sarcoma. Biphasic CT (liver window) demonstrates marked arterial hyperenhancement of lesions in Segment III and V (**a**, white arrows). These demonstrate rapid washout in the portal venous phase (**b**, black arrows) in keeping with hypervascular metastases

**Fig. 6:**
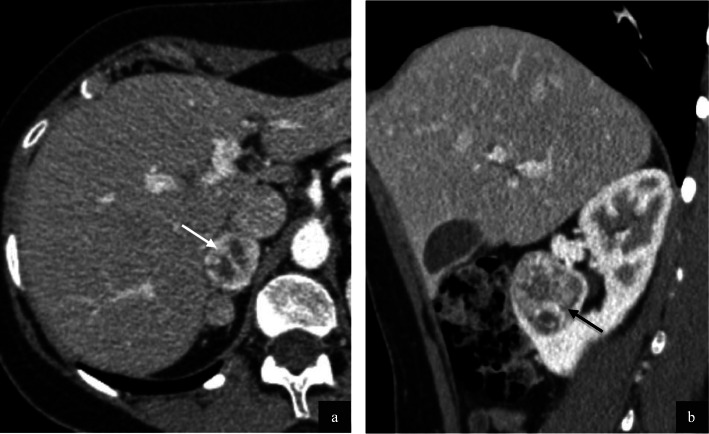
Adrenal (**a**) and renal (**b**) metastases in a 21 year old female with alveolar soft part sarcoma. Portal venous phase contrast enhanced axial (**a**) and sagittal (**b**) CT demonstrates a lobulated, hyperenhancing right adrenal metastasis (**a**, white arrow) with central necrosis. The enhancing, right lower pole renal metastasis (**b**, black arrow) shows minimal necrosis

With regard to intrabdominal metastases, liver and splenic metastases were observed most commonly in 6/32 patients (19%) and renal and adrenal metastases in 4/32 (13%). Hyperenhancement was observed in hepatic and renal metastases (Figs. 5 and 6).

**Fig. 7:**
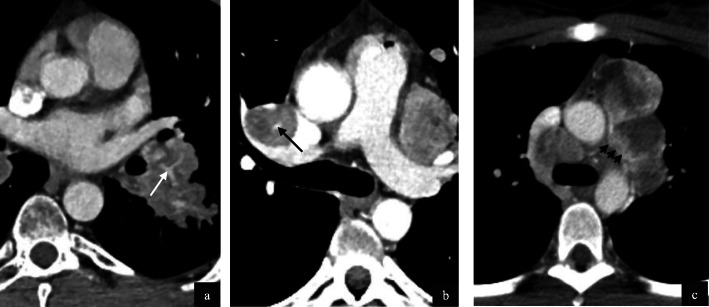
Contrast enhanced axial CT in three patients with nodal metastases from primary ASPS (**a**-**c)**. Left hilar nodal tissue in a 31 year old male patient demonstrates high attenuation with prominent central vessels (**a**, white arrow). Feeding vessels are demonstrated alongside and within nodal disease in a 37 year old male patient (**b**, black arrow). Metastatic mediastinal nodal disease in a 28 year old female (**c**) demonstrates marked central necrosis, however peripheral hypervascularity is still appreciated (**c**, black arrowheads)

Lymph node metastases were identified in 7/32 patients (22%), with 5 mediastinal metastases and 1 mediastinal with pelvic side-wall. All nodes demonstrated necrosis and hypervascularity, (Fig. 7) with prominent feeding vessels identified in 4/7 (57%).

**Fig. 8:**
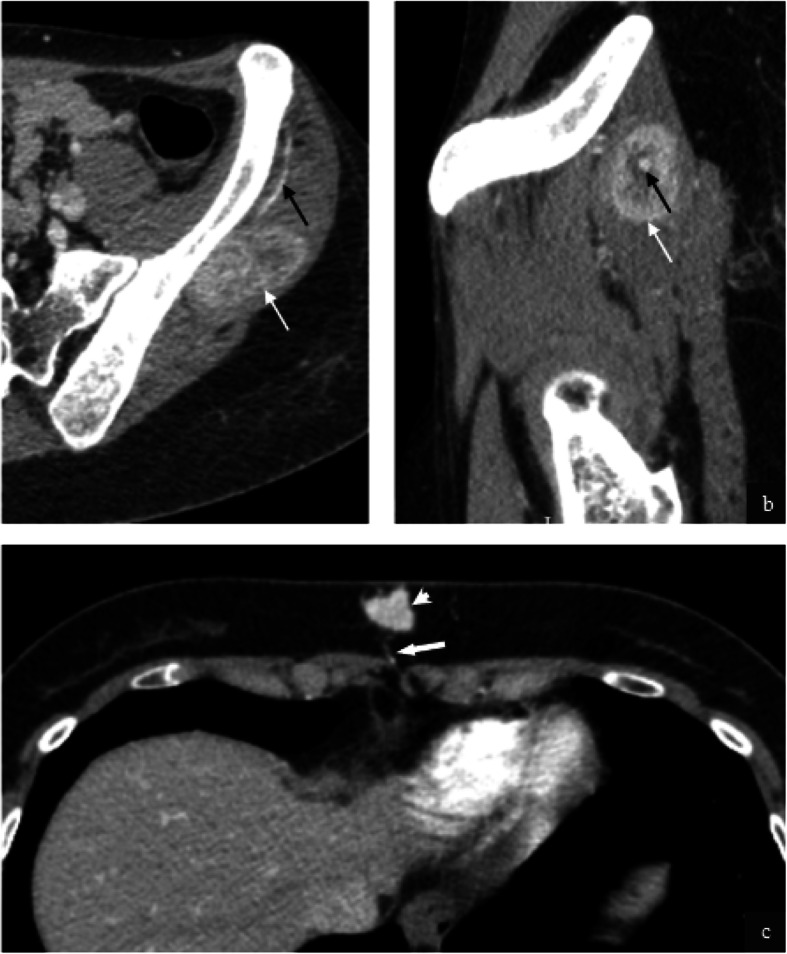
Intramuscular and subcutaneous metastases from ASPS. Post contrast axial (**a**) and sagittal CT (**b**) in a 21 year old female with an intramuscular metastasis (**a** & **b**, white arrows) demonstrates a large feeding vessel (**a** & **b**, black arrows). Post contrast axial CT in this 28 year old female patient demonstrates markedly hyperenhancing subcutaneous metastasis (**c** white arrowhead) inferior to the xiphisternum. Again a small feeding vessel is appreciated (**c**, thick white arrow)

**Fig. 9:**
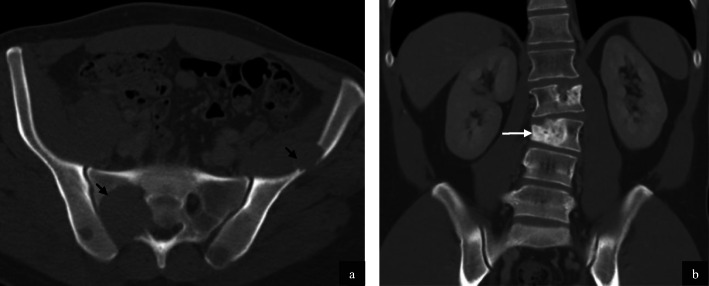
Bone metastases in two patients with alveolar soft part sarcoma. Axial CT of the pelvis on bone windows demonstrates multiple lytic metastases in this 69 year old male patient (**a**, black arrows). Coronal CT of the abdomen on bone windows in a 31 year old male patient demonstrates mixed sclerotic/lytic metastasis (**b**, white arrow) with associated with vertebral body collapse

Intramuscular (4/ 32, 13%) and subcutaneous metastases (1/32, 3%) were also featured. Metastases in these locations were characterised by hypervascularity with demonstrable feeding vessels (Fig. 8). The pattern of distant bone metastases (5/32, 13%) in our cohort was varied with lytic, mixed lytic and sclerotic and sclerotic-only metastasis appreciated (Fig. 9). Soft tissue components were observed in 3/5 cases (60%).

## Discussion

ASPS is characterised by a high rate of metastases, which can be present at diagnosis or occur during follow-up [8, 9] Whilst the imaging features of primary ASPS have been well described, less is known with regards to the imaging appearances of metastases. With 85% of patients developing metastases at any anatomic site up to 20 years after initial presentation, knowledge of these imaging appearances is useful for diagnosis, surveillance and further management.

Histopathological assessment of ASPS demonstrates marked tumour vascularity [10] with cytogenetic analysis showing upregulation of genes associated with angiogenesis [11]. These findings are concordant with the radiological appearance of primary ASPS. On angiography the presence of enlarged vessels with appearances suggestive of arteriovenous malformations have previously been described [12]. On MRI peri-lesional vessels with signal flow voids suggesting rapid blood flow, as well as central hypervascularity have also been demonstrated [13, 14]. High flow feeding and draining vessels have also been illustrated [15], occasionally assisting in differentiating this tumour from other sarcomas. Analysis of the imaging available for this prospective cohort confirms CT findings of primary ASPS in this group are consistent with those of primary ASPS described in the literature, namely, the presence of feeding vessels (100% of patients) and increased enhancement (100%).

Pulmonary metastases in our cohort were usually multiple with 75% of patients having more than 10 lesions. Within the original cohort, 47.9% of patients had received previous therapy prior to enrolment on the trial, confirming the difficulties in treating ASPS. Imaging features included contrast hyperenhancement (greater than skeletal muscle) in 83% (25/30 patients), whilst clearly defined feeding vessels were identified in 70% of cases (21/30). Studies by Portera et al. (2001) have commented on similar findings in pulmonary metastases with Choi et al 2000 [16] describing dilated tubular structures within the pulmonary masses which they suggest may represent vascular engorgement. Similar to previous ASPS series [17], other features of pulmonary involvement such as lymphangitis carcinomatosis were not observed.

Metastases to the brain have been described more commonly in ASPS [18, 19] than in other sarcomas, with an incidence of between 15 to 43% [20]. Neurologically stable patients with intracranial metastases were considered eligible for inclusion in CREATE, provided that they were not receiving steroids or additional contraindicated therapy [21]. The European Society for Medical Oncology/European Sarcoma Network Working Group suggests cranial imaging as part of the workup for ASPS [22]. However, having observed that brain metastases only occur in the context of metastatic disease and never in the absence of lung metastases, Portera et al. 2001 suggest that cranial imaging should be employed in symptomatic patients only. This observation is supported further by Kayton et al [23] and Malouf 2019 [24]. Although this is consistent with our findings, interpretation in our series is limited as intracranial imaging was only performed in 3/32 patients.

Metastatic, hyperattenuating lymph nodes were identified in 7/32 cases (22%) and necrosis within these pathological nodes was common (6/7; 86%), even in smaller lesions. The incidence of nodal involvement reported in the literature varies from 7 to 10% [25] to 75% [21]. Nodal metastases itself is a distinct feature of ASPS, with nodal involvement rarely observed in most other sarcoma subtypes. In addition the increased vascularity observed in relation to these nodal metastases in our cohort may represent an additional distinctive feature of ASPS metastases.

The presence of feeding vessels was less pronounced in metastatic disease involving solid abdominal viscera but these lesions remained hyperenhancing. Bone and soft tissue deposits were the third most common site of metastases. Appearances were variable with no defining features. Intramuscular and subcutaneous metastases unsurprisingly had features that closely resembled the primary tumour in all cases.

Primary ASPS has been described as containing calcification seen microscopically [26] and on plain film [27]. However, calcification was only observed within bone metastases and not at other sites of metastases. Haemorrhage at metastatic sites was also a notably absent feature; which was which was remarkable given the vascularity associated with imaged metastases.

Genetic profiling of patients with ASPS has demonstrated an upregulation of factors involved in angiogenesis such as Hypoxia-Inducible factor 1α, Tyrosine Kinase and Vascular Endothelial Growth Factor (VEGF) [28]. This upregulation of angiogenesis in ASPS has been associated with specific genetic translocations including over expression of the MET receptor. Targeted inhibition of this abnormal, overexpressed MET receptor with crizotinib has produced promising results within the CREATE trial. The employment of targeted molecular therapy has also led to positive outcomes with cediranib (an inhibitor of vascular endothelial growth factor receptor tyrosine kinases) as part of the CASPS trial [29]. Recognition and characterisation of these angiogenic features on imaging may offer an additional formal method of evaluating treatment response; not only quantitatively but also in terms vascularity of the primary tumour and its metastases.

Characterisation of the features of metastatic ASPS has value at diagnosis as many patients present with metastatic disease. Furthermore, in patients with previously resected tumour, metastasis can occur many years after resection. We have observed hypervascular metastases that have feeding vessels in the majority of cases, most commonly associated with pulmonary deposits.

## Conclusion

Imaging review of metastases from ASPS of patients within the CREATE study demonstrates that metastases are frequently hypervascular 94% (30/32) with prominent feeding vessels, most consistently defined in pulmonary metastases in 70% (21/30). These image findings in metastatic disease mirror previously described imaging appearances of primary ASPS in the literature.

## Data Availability

The data that support the findings of this study are available from “EORTC data sharing” but restrictions apply to the availability of these data, which were used under license for the current study, and so are not publicly available. Data are however available from the authors upon reasonable request and with permission of “EORTC data sharing”.
